# Reliability of the AR-CALUX^®^*In Vitro* Method Used to Detect Chemicals with (Anti)Androgen Activity: Results of an International Ring Trial

**DOI:** 10.1093/toxsci/kfab078

**Published:** 2021-06-24

**Authors:** Anne Milcamps, Roman Liska, Ingrid Langezaal, Warren Casey, Matthew Dent, Jenny Odum

**Affiliations:** 1 Joint Research Centre, European Commission, Ispra, Italy; 2 NIEHS, NTP, RTP, North Carolina, USA; 3 Unilever, Safety and Environmental Assurance Centre, Bedford, UK; 4 Independent Consultant, Macclesfield, UK

**Keywords:** endocrine disruption, androgen receptor, validation, specificity test

## Abstract

The AR-CALUX**^®^** *in vitro* method is a reporter gene-based transactivation method where endocrine active chemicals with androgenic or anti-androgenic potential can be detected. Its primary purpose is for screening chemicals for further prioritization and providing mechanistic (endocrine mode of action) information, as defined by the Organisation of Economic Cooperation and Development (OECD) conceptual framework for the testing and assessment of endocrine-disrupting chemicals. This article describes the conduct and results of an international ring trial with 3 EU-NETVAL laboratories and the test method developer. It was organized by EURL ECVAM to validate the method by testing 46 chemicals. A very good reproducibility within and between laboratories was concluded (94.7–100% and 100% concordance of classification) with low within and between laboratory variability (less than 2.5% CV on EC_50_ values). Moreover, the variability is within the range of other validated, mechanistically similar methods. In comparison to the AR-reference list compiled by ICCVAM, an almost 100% concordance of classifications was obtained. This method allows the detection of the agonist and antagonist properties of a chemical. A specificity control test was developed during the validation study and added to the antagonist assay rendering the assay more specific. A comparison is made with the mechanistically similar methods AR-EcoScreen™ and 22Rv1/MMTV GR-KO TA. The AR-CALUX^®^ method was approved for inclusion in the recently updated OECD test guideline TG458 which incorporates all 3 methods.

An endocrine disrupter (ED) is an exogenous substance or mixture that alters function(s) of the endocrine system and consequently causes adverse health effects in an intact organism, or its progeny, or (sub)populations (WHO/IPCS, 2002). Endocrine disruption may arise through many different ways but the main focus for many years has been on the disruption of the estrogen, androgen, thyroid hormone, and steroidogenesis pathways or modalities (OECD, 2018). Since 1996, a body of evidence has emerged that EDs may cause diseases in humans and animals such as endocrine-related cancers and reproductive disorders (WHO, 2013).

Significant progress in understanding and regulating EDs has been made worldwide over the past 20 years. Research, methods of detection, screening strategies, national and international guidance, criteria for the determination of ED properties, etc. have been developed and are subject to continuous revision and updating. The European Union put in place a Community Strategy for Endocrine Disruptors in 1999 (EC, 1999) which was revisited in 2018 (EC, 2018). The need for methods to identify EDs was also acknowledged by the Organisation of Economic Cooperation and Development (OECD) in the 1990s. In order to minimize exposure to EDs, standardized methods to reliably identify them are of utmost importance. Since 1996 the OECD has supported the validation of many standardized *in vitro* and *in vivo* methods for detecting EDs, their development into Test Guidelines (TGs) as well as guidance on their interpretation (OECD, 2020a).

Although by themselves reporter gene assays cannot identify a substance as an ED, they can identify the potential for endocrine activity *in vivo* which can be used in the identification of EDs by measuring biological activity upstream of the adverse effects that may be caused *in vivo.* Amongst the OECD approved TGs detecting chemicals with ED potential, there are currently 4 TGs, which include *in vitro* methods that investigate the estrogen, androgen, or steroidogenesis modalities; a steroidogenesis assay (TG456: OECD, 2011); an estrogen-receptor binding assay (TG493: OECD, 2015); an estrogen receptor transactivation assay (TG455: OECD, 2016); and an androgen receptor transactivation assay (TG458: OECD, 2020b). 

This article describes the conduct and results of an international ring trial of an AR-CALUX^®^ transactivation assay that has recently been included in the updated TG458 of Androgen Receptor TransActivation assays (ARTA) (Androgen Receptor TransActivation Assays for Detection of Androgenic Agonist and Antagonist Activity of Chemicals using Stably Transfected Cell Lines; OECD, 2020b). The original Test Guideline from 2016 described the AR-EcoScreen™ assay and the 2020 update include a further 2 newly validated assays: the ARTA described here and another mechanistically similar assay (the 22Rv1/MMTV-GR-KO assay) (Milcamps *et al.*, 2020; Park *et al.*, 2021). Both ARTA assays contain reporter genes, which are activated by the Androgen Receptor (AR) when bound to a ligand, ie, a chemical with androgen receptor affinity. This receptor-ligand complex enters the nucleus where it will bind to specific recognition sequences in the promoter region of a target gene (so-called androgen responsive elements or ARE). Hence, the target gene will be transcribed. When the target gene expresses the reporter (luciferase), *in vitro* hormonal activity of chemicals can be quantified as relative light units by luminescence measurement as well as the agonistic or antagonistic mode of action determined.

The AR-CALUX^®^ cell line was identified within the European Union funded project ReProTect (LSHB-CT-2004-503257) which aimed at optimizing an integrated set of tests as a basis for a reproductive/developmental battery, in order to provide a detailed understanding of the main tissues or biological mechanisms which could be targeted and disrupted by toxicants across different stages of reproduction. The parental line of the AR-CALUX^®^ cell line is the human osteosarcoma cell line U2-OS (ATCC HTB 96). This cell line stably expresses the human AR, is highly selective in its response to low levels of different androgens (due to the multimerized ARE and a minimal promoter-TATA box only), and has an insignificant response to other nuclear hormone receptor ligands such as estrogens and glucocorticoids (due to the cells not expressing other steroid receptors that can activate transcription via the same ARE as the androgen receptor) (Sonneveld *et al.*, 2005). The AR-CALUX^®^ method can be up-scaled for use on a robotic platform for high throughput screening purposes (Van der Burg *et al.*, 2015). The test method developer of this method carried out a pre-validation (van der Burg *et al.*, 2010) and subsequently submitted the method to EURL ECVAM for validation. EURL ECVAM accepted the submission and a formal validation study was initiated with the involvement of the European Network of laboratories for the validation of alternative methods (EU-NETVAL). This ring trial constituted the first validation exercise of the expert laboratory network EU-NETVAL, which was established in 2014 with the aim of supporting EURL ECVAM in its validation studies and related activities.

## MATERIALS AND METHODS

###  

#### Organization of the Validation Study

##### Coordination and Management

EURL ECVAM coordinated the validation study, starting with a call for interest directed to the EU-NETVAL, followed by the selection and approval of 3 participating laboratories. A Validation Management Group (VMG) was established to support the coordination which included the design of the validation study, the guidance, and facilitation of the validation process, evaluation of the data during the study, assistance with troubleshooting, approval of the chemical selection, analysis, and conclusion on the outcome. The VMG AR-CALUX^®^ was comprised of 3 experts in the field: Warren Casey, Matthew Dent, Jenny Odum, as well as Anne Milcamps (coordinator), and Roman Liska (statistician).

##### Participating Laboratories

Following the above-mentioned Call for Interest, the following 3 Good Laboratory Practices (GLP) laboratories were selected to participate in the trial on the basis of pre-defined criteria. The laboratories were Research Institutes of Sweden (RISE, formerly named SP Technical Research Institute, Sweden), Charles River Labs (formerly named CitoxLAB, France), Covance (formerly named Huntingdon Life Sciences, then Envigo, UK). After approval of these 3 facilities by the National Contact Points, collaboration agreements were put in place. The test method developer BioDetection Systems (BDS, the Netherlands) acted as a fourth laboratory.

##### Validation Study Design

The study was initiated by training of the 3 EU-NETVAL labs followed by data generation according to the modular approach (Hartung *et al.*, 2004). Part 1 of the study (transferability) involved data generation for 6 test chemicals and Part 2 (within laboratory reproducibility (WLR) and between laboratory reproducibility (BLR)) required data from 20 test chemicals. An additional 24 test chemicals (Part 3) were analyzed by 1 lab only for the purpose of relevance evaluation. GLP compliance was required for Parts 2 and 3 studies. All chemicals for the study were provided by EURL ECVAM including blind coding for the chemicals used in Parts 2 and 3. The statistical analysis of the data was performed by the statistician of the VMG.

The validation study was organized according to OECD Guidance Document 34 (OECD, 2005) and 3 independent runs (ie, 3 different cell batches tested at different times) that met the acceptance criteria (valid runs) were required. This included 3 prescreen runs when a negative response was observed, or, 1 prescreen run followed by 3 comprehensive runs for an observed positive response. While a prescreen run would have a dilution series of 1:10, a comprehensive run had a dilution factor of 3 in order to better define the concentration response. All test chemicals were assessed with both an agonist and antagonist assay. For the antagonist assay, a specificity control test was included. A quantitative cytotoxicity test (Lactate Dehydrogenase (LDH)) was included for each agonist and antagonist assay, for the initial prescreen run and the second prescreen run (in case of a negative response), or, for the initial prescreen run and the first comprehensive run (in case of a positive response). For all runs (prescreen and comprehensive), the cytotoxicity of the cells was evaluated visually with a microscope.

#### Test System

The AR-CALUX^®^ cell line was created by BioDetection Systems (BDS). It can be purchased from BDS with a license (https://www.biodetectionsystems.com). The human osteosarcoma cell line U2-OS (ATCC HTB 96) was transfected with 2 constructs: pSG5-neo-hAR carrying the cDNA of a human androgen receptor (hAR) under a constitutive promoter, and, a luciferase reporter gene which is preceded by a triple tandem of AREs in front of a TATA box (3x ARE Luc). A detailed description can be found in Sonneveld *et al.*, 2005. The cell line had been reported to be highly selective in its response to low levels of androgen and to have an insignificant response to other nuclear hormone receptor ligands such as estrogens and glucocorticoids (Sonneveld *et al.*, 2005). The AR-CALUX^®^ cell line was obtained from the test method developer BDS. EURL ECVAM prepared a master cell bank and a large working cell bank which were tested for the absence of mycoplasma, bacteria, fungi, and yeast and for the absence of Hepatitis B and C and HIV. In addition, to verifying the authenticity of the cell line, Short Tandem Repeat (STR) profiling was carried out to confirm the test system to be U2-OS cells. With STR profiling nuclear DNA is analyzed for the presence of 8 or more different and highly polymorphic short tandem repeat (STR) loci according to the ANSI/ATCC ASN-0002-2011 standard “Authentication of Human Cell Lines: Standardization of STR Profiling.” Aliquots of the working cell bank were distributed by EURL ECVAM to the participating laboratories for the different parts of the study. The laboratories were requested, at the end of each part of the study (Parts 1, 2, and 3), to perform a mycoplasma test of their choice. Additional aliquots were sent to EURL ECVAM for a reanalysis of authenticity.

#### Reference, Control, and Test Chemicals

Reference and control chemicals applied to confirm the correct functioning of the cells and validity of the experiments are listed in Table 1. The full list of reference, control and test chemicals used in the validation study is shown in Supplementary Table 1.

**Table 1.** Reference and Control Chemicals for the Agonist Assay and Antagonist Assay

	Name	CAS No.
**Agonist**		
Reference	Dihydroxytestosterone (DHT)	521-18-6
Positive control	Methyl testosterone	58-18-4
Negative control	Corticosterone	50-22-6
**Antagonist**		
Reference	Flutamide	13311-84-7
Positive control	Linuron	330-55-2
Negative control	Levonorgestrel	797-63-7

#### Test Chemical Selection and Distribution

An initial list of 83 possible chemicals to be tested was compiled by EURL ECVAM in 2015 based on the available literature, the ICCVAM recommendation for ARTAs (ICCVAM, 2003), data obtained in 2 Tox21 ARTA methods (Tox21 AR-BLA based on human embryonic kidney cells (HEK293T) and Tox21 AR-luc based on human breast carcinoma cells (MDA-kb2), QSAR data (Pass AR agonist model (http://www.pharmaexpert.ru/passonline/) and expert consultation (OECD’s Validation Management Group non-animal (VMG-NA) and the International Cooperation on Alternative Test Methods (ICATM)). In addition, the chemicals were tested in the EURL ECVAM laboratory via high throughput screening for a first evaluation (without cytotoxicity testing). A subset of 45 chemicals was selected, aiming at a balanced set of agonist, antagonist, and negative chemicals. For details on the selection see Annex 13.4 of the Validation Study report (Milcamps *et al.*, 2020). Briefly, the criteria for selection were as follows: potency as an AR agonist/antagonist, classification as an agonist/antagonist according to the group performing the chemical selection, good availability and price, known properties, structural diversity, and chemical space distribution. When the test chemicals of the ongoing OECD validation studies of 2 mechanistically similar methods, the AR-EcoScreen™ method and the 22Rv1/MMTV GR-KO method, became available, the lists were compared. Those not yet present in the validation set for the AR-CALUX^®^ method were added. In addition, a putative false antagonist chemical was added as a control for the specificity control test in order to identify non-competitive binding at the AR. The chemical was selected based on Tox21 AR-luc data showing antagonist activity at non-cytotoxic concentrations but considered to be a non-specific reaction (VMG AR-CALUX^®^). In 2017, the ICCVAM AR-reference list was published, based on an extensive literature and data search for AR binding and transcription assay, followed by rigorous selection based on a set of quality criteria (Kleinstreuer *et al.*, 2017). In comparison with this list, a few positive antagonist chemicals were added to the AR-CALUX^®^ validation set given the limited number of this type of test chemicals. In total, 53 chemicals were selected by the VMG AR-CALUX^®^ to be tested in the validation study covering Part 1 (6 chemicals), 2 (20 chemicals), and 3 (24 chemicals) with only 2 chemicals used in more than 1 part and the positive control (PC) and the negative control (NC) also used as test chemicals. For all phases in the validation study, chemicals were chosen as such to cover an even distribution of expected positive and negative classifications for agonist and antagonist assays, based on reported data.

The chemicals were purchased from Sigma-Merck (Italy), TCI Europe (Belgium), Chemos (Germany), Carbosynth (UK), and LGC Standards (Italy) and distributed to the participating labs. The test chemicals for Parts 2 and 3 were blind coded. The identity of the test chemicals was released only when all experiments were completed and the data analysis of blind coded chemicals had been finalized by the statistician.

#### Standard Operating Procedures and Data Analysis Files

Prior to the start of the study, the test method developer provided draft Standard Operating Procedures (SOPs) to EURL ECVAM. Both parties further developed and optimized technical details in the SOPs such as the formulation of acceptance criteria for a run, development of a specificity control test plus criteria, and the formulation of a classifier to determine an agonist and an antagonist. A first version of the draft SOP combining agonist and antagonist assays was experimentally assessed in the EURL ECVAM laboratory for completeness, and, for its applicability in a GLP environment. Further optimizations followed.

The final SOP for agonist and antagonist testing can be found on EURL ECVAM’s Tracking System for Alternative methods towards Regulatory acceptance (TSAR) (EURL ECVAM, 2020a). It details the cell cultivation and seeding procedure, the preparation of the reference, control and test chemicals, the procedure for agonist testing and antagonist testing, prescreen runs, and comprehensive runs, as well as data analysis and data interpretation. This SOP also includes references to the SOPs for solubility testing and cytotoxicity testing. EURL ECVAM provided an SOP for chemical solubility determination, based on visual inspection, centrifugation, and light microscopy for solubility observation (EURL ECVAM, 2020b). Solubility was tested both in the solvent and in media under similar circumstances as would be required during the (ant)agonist testing. The initial concentration to be tested was prescribed at 50 mg/ml. Starting from the highest soluble concentration, cytotoxicity was determined with an LDH assay. Visual checking of cytotoxicity in all wells of the plate was however also required for each run.

For the data analysis, files were provided in the form of Excel templates where the acceptability criteria to obtain valid runs are automatically calculated and graphs of dose responses are visualized. They can be found on EURL ECVAM’s TSAR (EURL ECVAM, 2020a).

#### Agonist Assay, Antagonist Assay, Specificity Control Test

Each test chemical was firstly tested at the highest soluble concentration, starting from 50 mg/ml. Subsequently, the test chemical was assessed with a prescreen run (dilution steps of 10) and simultaneously with the quantitative cytotoxicity test to determine (1) cytotoxic concentrations, (2) the response, and (3) the appropriate dose range to be tested. The level of unacceptable cytotoxicity was 15% LDH leakage or a morphological change observed by the microscope. In the following comprehensive test, only concentrations that did not show cytotoxicity were used and a dilution step of 3/3.3 was applied for more accurate determination of the parameters. The cytotoxicity test was performed once more. Test chemicals were diluted in media and consisted of 8 serial dilutions. Each dilution was tested in triplicate.

Each experiment (run) was comprised of a maximum of 6 plates. The first plate would contain 8 serial dilutions of the reference chemical DHT (for agonism) or Flutamide (for antagonism), while the positive and negative controls were added at fixed concentrations. In addition, a solvent control was added (medium without the chemical but with DMSO 0.1%).

**Figure 1.**
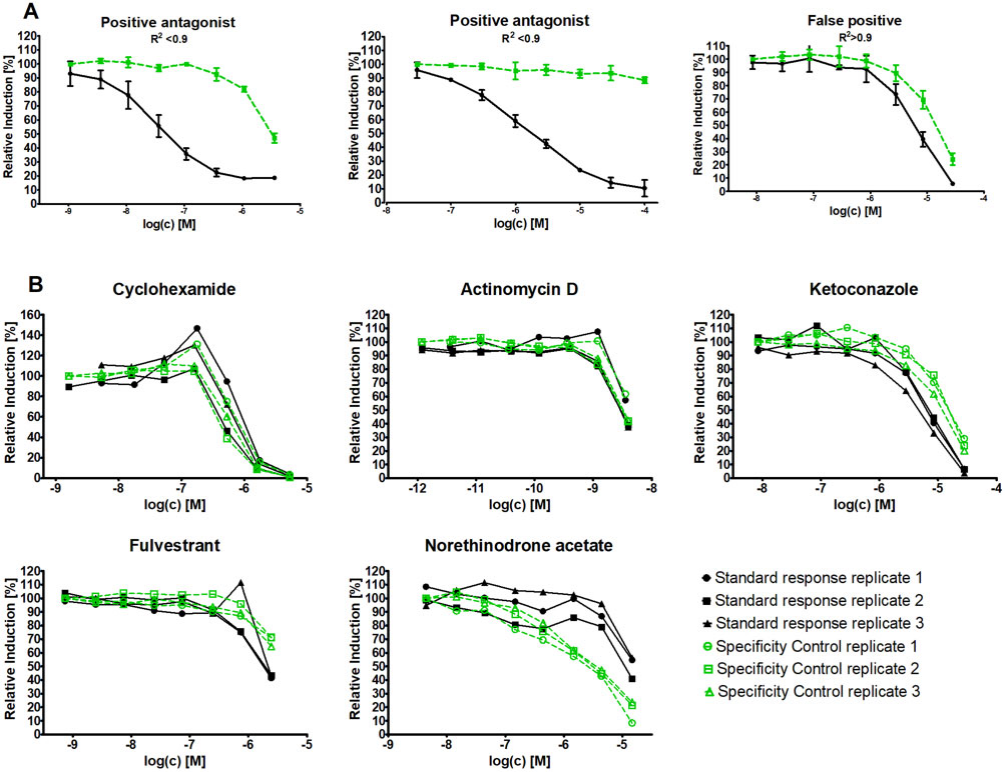
Dose responses obtained for competitive and false competitive antagonists. Solid line, standard response with medium spiked at EC_50_ DHT; dashed line, specificity control response with medium spiked at 100× EC_50_ DHT; dotted line indicates 80% Relative induction. Dose responses of 3 individual runs in 1 lab are presented. They have been normalized. *R*^2^ is the correlation coefficient, indicating the competitive (*R*^2^ < 0.9) or the false competitive antagonist (*R*^2^ > 0.9). A, Examples of a positive antagonist and a false antagonist. B, False antagonists observed in the AR-CALUX^®^ validation study. (*) indicates an outlier which was removed for the calculation of the *R^2^*.

For the antagonist assay, the medium was supplemented with DHT at EC_50_ concentration (standard assay). The vehicle control was comprised of DHT at EC_50_ while the solvent control was the VC with DMSO 0.1%. Each antagonist assay was accompanied by a specificity control test carried out on the same plate. This assay was carried out in a similar way as the standard assay except that the medium was supplemented with an excess of DHT (100× EC_50_ DHT). This leads to 2 dose responses which for a positive antagonist are clearly separated (excess DHT shifted to the right) as opposed to overlapping in the case of a false positive antagonist. The shift can be quantified via the correlation coefficient *R*^2^ (Figure 1A; Liška *et al.*, 2021).

#### Assay Acceptability Criteria and Data Interpretation Criteria

Criteria for an experiment (run) to be valid were developed by the test method developer and EURL ECVAM and discussed with the AR-CALUX^®^ VMG. For Part 2 of the study (WLR and BLR), the specificity control test was included. Hence, an additional criterion was incorporated for acceptance of the antagonist assay data. A list of these criteria can be found in Table 2. Test method developer, EURL ECVAM, and AR-CALUX^®^ VMG also established the criteria for the data interpretation leading to a classification of positive or negative (see Supplementary Table 2).

**Table 2.** Acceptability Criteria

Agonist Assay			Antagonist Assay		
No.	Criterion	Value	No.	Criterion	Value
	Reference chemical DHT			Reference chemical Flutamide	
1	Curve fitting	Sigmoidal	1	Curve fitting	Sigmoidal
2	EC_50_ range	1.0×^−10^ to 1.0×^−9^ M	2	IC_50_ range	1.1×^−7^ to 1.1×^−6^ M
3	CV of estimated log(EC_50_)	<1.5%	3	CV of estimated log(IC_50_)	<3%
4	Induction factor	>20	4	Inhibition factor	>10
5	Z-factor	>0.5	5	Z-factor	>0.5
	Positive control			Positive control	
6	RI methyl testosterone	>30%	6	RI Linuron	<60%
	Negative control			Negative control	
7	RI corticosterone	<10%	7	RI levonorgestrel	>85%
				Reference chemical Flutamide specificity control	
			8(*)	*R* ^2^ between the RI of Y_c_ and S_c_ for Flutamide	≤ 0.7

Abbreviations: RI, relative induction; *R^2^*, the square of the correlation coefficient. (*) only for specificity control.

#### Data Collection, Analysis, and Statistics

All raw data obtained by the participating labs were transferred to and automatically analyzed in data analysis files (Excel templates). These forms had been developed by the test method developer and EURL ECVAM, validated by EURL ECVAM, and provided to the participating labs. These forms, once compiled, were quality controlled by the participating labs and uploaded on the EU platform for data exchange: CIRCABC. Data were retrieved and reanalyzed by the EURL ECVAM statistician, and, compared across all 4 labs. All data, including those obtained from the specificity control test, were normalized with respect to the reference chemicals DHT (for agonism) or Flutamide (for antagonism). The data that fulfilled the acceptance criteria (ie, valid runs) were used to evaluate overall variability of the obtained data, to verify the specificity of test chemicals with antagonist activity, to determine reproducibility within laboratories (WLR) and between laboratories (BLR), calculate performance values (eg, specificity, sensitivity, etc.) and assess the concordance of classifications with the ICCVAM AR-reference list. The data were visually presented with dose responses (Supplementary Figures 1, 2, and 3). Evaluation of the relevance of the classifications obtained was carried out by comparison with publicly available sources of classifications from *in vitro* methods, ie, the ICCVAM AR-reference chemical list (Kleinstreuer *et al.*, 2017), the results of 2 validated ARTA methods (TG458 and reports) and a list compiled by EURL ECVAM based on publicly available data (Milcamps *et al.*, 2020) which includes the classifications of 2 Tox21 ARTA assays (US EPA) and the results of the AR pathway computational model (Kleinstreuer *et al.*, 2017).

## RESULTS

###  

#### Test Method Definition Including Development of the Specificity Control Test

A draft SOP had been received from the test method developer to which EURL ECVAM provided additional parts and modifications. In collaboration with the test method developer, the different aspects were expanded in description and detail, aiming at an unambiguously understandable SOP. The acceptance criteria were further developed, with the inclusion of the induction factor (IF) per plate, and the Z-factor per plate. These 2 parameters were calculated on the basis of the values for the reference chemical (DHT or Flutamide, at a fixed concentration on each plate) and the background (solvent control on each plate).

A specificity control test for antagonism was added allowing the confirmation of a true competitive action at the binding site of the androgen receptor. Such a test had been recommended by the VMG AR-CALUX^®^ given the non-specific responses observed in other methods eg in the ToxCast programme eg the antagonist luciferase assay in the MDAKB2 cell line (A11) (Kleinstreuer *et al.*, 2017). These nonspecific responses may be due to cytotoxicity which is not detected due to the absence of a cytotoxicity test in the antagonist assay, or use of a cytotoxicity test which is less sensitive or reports only on the final events of cytotoxicity eg cell wall disruption. Other factors may also influence the response of the chemical eg interference with the signal generation pathway at the level of cofactor aggregation, ARE binding, transcription, translation, stability of the luciferase enzyme, signal activity (Borrel *et al.*, 2020a,b). The specificity control test described in this article consists of 2 assays, the standard antagonist assay where the medium is spiked with the EC_50_ concentration of the ligand DHT, and, a parallel assay in which the medium is spiked with a higher concentration of the ligand DHT (100× EC_50_). The higher ligand concentration results in a greater competition of DHT with the test chemical in binding to the AR, leading to a delayed, ie, shifted dose response (see Figure 1A). A false antagonist however shows overlapping dose responses (normalized) indicating that the observed decrease in signal is not linked to binding to the receptor. In the AR-CALUX**^®^** method, a quantification of the shift was introduced via the correlation coefficient *R*^2^, where the similarity of the effect with the 2 concentrations of DHT at a given concentration of test chemical is compared (Liška *et al.*, 2021). On the basis of in-house data from EURL ECVAM and BDS, the threshold for criterion *R*^2^ was set at 0.7 for the reference chemical Flutamide (<0.7 means acceptance of the run) and 0.9 for a test chemical (<0.9 means confirmation of the antagonist response).

For the data interpretation, a classifier was developed to define a positive or negative assessment for each chemical when tested with the agonist or antagonist assay (Supplementary Table 2). For antagonism classification, the result of the specificity control test was taken into account to come to a final conclusion.

Prior to the initiation of the experimental part by the participating labs, the SOP was assessed at EURL ECVAM for completeness, clarity, and application under GLP conditions. It was experimentally assessed in the laboratory with a few chemicals to perfect the SOP.

#### Chemical Selection and Chemical Space Analysis

A collection of 53 chemicals was compiled (see Materials and Methods and Supplementary Table 1) which included the reference chemicals DHT and Flutamide (not used as test chemicals), the control chemicals, and the test chemicals. The 6 chemicals used in the transfer part of the validation study were not used again for the other parts in order to avoid a bias due to gained familiarity with these chemicals. One exemption was sodium azide, which was used again in Part 2, given that it had led to some challenges in Part 1. Forty-four chemicals were retained for testing in the WLR, BLR, and relevance part of the validation study. The negative and positive controls, which were used at fixed concentrations in agonist and antagonist assays, were also used as coded test chemicals for a dose response evaluation. To assess BLR, 20 chemicals were tested by all 4 labs; to assess relevance an additional 24 chemicals were tested by 1 lab only.

The structural space of the chemicals was investigated based on structural similarity and cluster analysis (Avalon structural fingerprint, Tanimoto similarity). Chemicals had been chosen on basis of maximum structural diversity. Mapping of the selected chemicals versus the REACH chemical space showed a good distribution (Supplementary Figure 4).

#### Training

Prior to the initiation of the experimental work, representatives from all participating laboratories attended a training session on the critical steps of the method. Training was provided by EURL ECVAM and the test method developer at EURL ECVAM’s premises. Hands-on training in the laboratory was provided as well as reporting and analyzing data using the provided data analysis forms (Excel sheets). The European Commission CIRCABC platform for data exchange was introduced to the laboratories.

#### Transferability

The first part of the validation study consisted of the implementation of the method by the labs in their own facility and assessment of the ability to carry this out in a correct manner. Six non-coded chemicals were tested with the agonist and antagonist assay (Supplementary Table 1). Cytotoxicity testing was required. Three valid runs (ie, runs where all acceptance criteria had been met) were mandatory and classification was not yet requested. EURL ECVAM evaluated the results through comparison with data obtained in EURL ECVAM’s facility, assessing the obtained parameters for eg potencies and the obtained dose responses. After some initial technical issues, the labs were considered as being sufficiently familiar with the method in order to perform experiments. Some examples of the technical issues encountered were as follows. 2 labs picked up endocrine activity from either the glass tubes used for making dilutions or from glass/plastic ware used during the preparations. The issue was remediated by changing the brand of the glass tubes and testing all material up front for endocrine activity. One lab experienced occasionally too low or too high Relative Light Units (RLUs), which was traced back to the use of too highly concentrated preparations of Triton x100 (cytotoxicity control) that affected the nearby wells or to incorrect temperature of luciferin. One lab experienced parameter issues with the luminometer. Given the lab’s expertise with a luciferase kit other than that referred to in the SOP, remediation was achieved by using the familiar kit. Based on the encountered experiences by the labs, technical adjustments to the SOP were implemented in order to avoid different interpretations, and, to encompass a broader range of reagents to measure luminescence.

#### Solubility and Cytotoxicity

For solubility testing, an SOP was provided by EURL ECVAM based on visual inspection (EURL ECVAM, 2020b). This included an investigation by heating, centrifugation, microscopy, and visual checking. The labs were asked to find for each of the test chemicals: the appropriate solvent; the highest solubility in the chosen solvent starting from 50 mg/ml; the highest solubility in assay medium (Supplementary Table 3). All 4 labs reported slightly different soluble concentrations. One lab was more conservative, with consistently lower maximum solubilities recorded in its observations and determinations though this did not affect the outcome of the study. For the purpose of the validation study, the labs were requested to start from 50 mg/ml as the maximal stock concentration to be tested. Converted to molarity, the range for the chemicals tested would be between 40 mM and 330 mM with a few chemicals at a higher concentration eg 769 mM for Sodium Azide.

Cytotoxicity was evaluated in both the agonist and antagonist assay and was carried out with the LDH test which can be performed in parallel to the agonist/antagonist assay and for which no extra plates have to be prepared. A drawback of this test is that the measurement will occur when leakage happens, ie, when the cell wall is degrading and thus the cell is dead. Early events in the onset of cell death cannot be measured with this test. For this reason, the AR-CALUX**^®^** method stated that the cytotoxicity of the exposed cells should also be assessed visually (microscopy). It was found that the visual inspection was often more sensitive than the LDH kit.

#### Within Laboratory Reproducibility

WLR was assessed on the data generated by 20 test chemicals, obtained from 3 valid runs. Calculation of various parameters such as the EC_50_, PC_10_, IC_50_, and PC_80_ values for each run, the mean, SD, and CV per test chemical, and per lab can be found in the Validation Statistical report (Liška, 2020). Table 3 shows an overview of the CV for obtained values of logEC_50_ and logIC_50_ values per chemical and per lab. These CVs were low and generally below 4% (between 0.8% and 6.4% for the agonist assay and between 0.1% and 3.9% for the antagonist assay) while the CV values for the reference chemicals were between 0.69% and 2.72%. Potency values for each chemical are also shown in Table 3.

**Table 3.** Within Laboratory Variability per Chemical, Potency per Chemical, and Between Laboratory Variability for All Chemicals, Tested with Agonist and Antagonist Assay

Agonist (logEC_50_)							Antagonist (logIC_50_)					
Test Chemical	Lab 1 CV	Lab 2 CV	Lab 3 CV	Lab 4 CV	Overall logEC_50_	Overall CV	Lab 1 CV	Lab 2 CV	Lab 3 CV	Lab 4 CV	Overall logIC_50_	Overall CV
	Within laboratory variability						Within laboratory variability					
17β-Trenbolone	0.80%	0.54%	0.50%	0.32%	−9.61	0.9%						
Stanozolol	1.14%	1.54%	0.37%	0.84%	−8.76	0.8%						
Spironolactone							0.32%	0.78%	0.31%	0.37%	−7.44	0.2%
Medroxyprogesterone acetate	0.57%	6.42%	1.21%	1.01%	−8.16	4.0%						
Bisphenol A							0.97%	0.82%	0.15%	0.32%	−5.86	0.3%
Bicalutamide							1.64%	0.30%	2.77%	1.26%	−6.95	1.8%
Disulfiram												
Tamoxifen												
Atrazine												
17α-Ethynyl estradiol							1.45%	2.12%	0.90%	1.69%	−7.41	0.1%
Sodium azide												
Diethylhexyl phthalate												
Methyldihydrotestosterone	0.82%	0.47%	0.44%	0.28%	−9.47	0.3%						
Vinclozolin							0.32%	2.02%	0.47%	3.93%	−7.01	2.0%
Prochloraz							1.08%	1.44%	0.95%	0.62%	−5.66	0.5%
Fluoxymesterone	1.46%	1.82%	0.74%	1.00%	−7.82	3.4%						
17β-Estradiol							2.22%	2.30%	0.96%	0.98%	−8.05	2.1%
Benzylbutyl phthalate							1.97%	1.87%	1.56%	1.79%	−5.36	4.9%
Propylthiouracil												
Hydroxyflutamide							0.87%	0.57%	0.46%	0.83%	−7.69	1.0%
	Between laboratory variability						Between laboratory variability					
All test chemicals	0.96%	2.16%	0.65%	0.69%			1.20%	1.28%	0.95%	1.31%		

WLR: CV results from 3 valid runs per lab; overall CV results from CV calculated on the averaged logEC_50_ and logIC_50_ calculated per lab; blank spaces: no response. BLR: CV calculated on the averaged logEC_50_ and logIC_50_ of all test chemicals.

Evaluation of WLR via the application of the classification (Table 4) showed an almost 100% concordance within each lab for both agonist and antagonist testing.

**Table 4.** Classifications Obtained Per Run in 4 Labs for 20 Chemicals Tested with Agonist and Antagonist Assay

	Agonist				Antagonist			
Test chemical	Lab 1	Lab 2	Lab 3	Lab 4	Lab 1	Lab 2	Lab 3	Lab 4
17β-Trenbolone	P P P	P P P	P P P	P P P	N N N	N N N	− N N	N N N
Stanozolol	P P P	P P P	P P P	P P P	N N N	N N N	N N N	N N N
Spironolactone	N N N	N N N	N N N	N N N	P P P	P P P	P P P	P P P
Medroxyprogesterone acetate	P P P	P P P	P P P	P P P	N + N	N N N	N N N	N N N
Bisphenol A	N N N	N N N	N N N	N N N	P P P	P P P	P P P	P P P
Bicalutamide	N N N	N N N	N N N	N N N	P P P	P P P	P P P	P P P
Disulfiram	N N N	N N N	N N N	N N N	N N N	N N N	N N N	N N N
Tamoxifen	N N N	*N N	N N N	N N N	N N N	N N N	N N N	N N N
Atrazine	N N N	N N N	N N N	N N N	N N N	N N N	N N N	N N N
17α-Ethynyl estradiol	N N N	N N N	N N N	N N N	P P P	P P P	P P P	P P P
Sodium azide	N N N	N N N	N N N	N N N	N N N	N N N	N N N	N N N
Diethylhexyl phthalate	N N N	N N N	N N N	N N N	N N N	N N N	N N N	N N N
Methyldihydrotestosterone	P P P	P P P	P P P	P P P	N N N	N N N	N N N	N N N
Vinclozolin	N N N	N N N	N N N	N N N	P P P	P P P	P P P	P P P
Prochloraz	N N N	N N N	N N N	N N N	P N P	P P P	P P P	P P P
Fluoxymesterone	P P P	P P P	P P P	P P P	N N N	N N N	N N N	N N N
17β-Estradiol	P P N	P P P	P P P	P P P	P P P	P P P	P P P	P P P
Benzylbutyl phthalate	N N N	N N N	N N N	N N N	P P P	P P P	P P P	P P P
Propylthiouracil	N N N	N N N	N N N	N N N	N N N	− N N	N N N	N N N
Hydroxyflutamide	N N N	N N N	N N N	N N N	P P P	P P P	P P P	P P P
WLR (%)	95	100	100	100	94.7	100	100	100
BLR (%)	100				100			

Abbreviations: N, negative classification; P, positive classification; WLR, within lab reproducibility; BLR, between lab reproducibility.

Chemicals not included in WLR assessment due to (*) disqualified run, (+) data difficult to analyze, (−) lack of specificity control.

For details see Liška (2020).

Grey shading indicates P classification of runs.

#### Between Laboratory Reproducibility

BLR was assessed on the same set of data from 20 chemicals that were evaluated for WLR. Reproducibility of the calculated parameters such as EC_50_, PC_10_, IC_50_, PC_80_ between the labs was evaluated. The range of % CV obtained for logEC_50_/logIC_50_ for all tested chemicals in this validation study is indicated in Table 3, being below 2.5% for both agonist and antagonist testing. This value is comparable to that reported for the ER-CALUX method (less than 3.5%) which was included in the TG455 and comparable to that reported for the AR-EcoScreen™ (adopted as TG458) and the 22Rv1/MMTV/GR-KO TA (see Table 5). In addition, BLR was assessed based on the concordance of classifications between the labs, applying the majority rule to arrive to 1 classification out of 3 valid runs per lab. BLR was 100% for both agonist and antagonist testing (Table 4).

**Table 5.** Observed Variability (CV) During the Validation Study from Different Methods

Validation Study	Agonist Testing	Antagonist Testing
AR-CALUX^®^	Log(EC_50_): 0.65–2.16% (5 test chemicals)	Log(IC_50_): 0.95–1.31% (9 test chemicals)
ER-CALUX^®^	Log(EC_50_): 1.2–3.1% (17 test chemicals)	Log(IC_50_): 0.5–1.6% (4 test chemicals)
AR-EcoScreen™	Log(PC_50_): 0.38% to 1.53% (3 test chemicals)	Log(IC_50_): 0.84–1.15% (3 test chemicals)
22Rv1/MMTV/GR-KO TA	Log(PC_50_): 3.54% (9 test chemicals)	Log(IC_50_): 2.85% (13 test chemicals)

CV determined on all obtained values for all tested chemicals per lab involved in the validation study.

The lowest and highest CV are indicated.

#### Specificity of the Antagonist Response

The inclusion of the specificity control test in the antagonist assay showed an added value to exclude false positives. Evaluation of a correct shift of the dose response of the specificity control (ie, to the right of the standard dose response) and calculation of the correlation coefficient *R*^2^ between the 2 dose response curves (standard vs specificity control) allowed classification of 5 out of 24 antagonist responses, ie, 20%, as nonspecific (for an overview of the 5 nonspecific antagonists, see Table 6 and Figure 1B). For the chemicals Cycloheximide, Actinomycin D, and Ketoconazole, 2 antibiotics, and a fungicide respectively the obtained *R*^2^ of > 0.9 indicated a false positive response. The non-specific behavior was likely due to cytotoxicity given the antibiotic/fungicide nature of the chemicals, which was not picked up by the LDH kit or microscopic observation. The chemical Fulvestrant with a *R*^2^ of > 0.9, is used in prostate cancer treatment and is thought to have its action by downregulating the expression of the androgen receptor (Bhattacharyya *et al.*, 2006). This could possibly explain the observed result of false positive in this study. With increasing concentrations of Fulvestrant, AR expression would decrease, with less AR available for binding with the ligand DHT.

**Table 6.** Criteria to Identify Specific versus Non-specific Antagonists with the AR-CALUX^®^ Specificity Control Test

Test Chemical	Shift Direction	$S_{c}^{n}$ (%)	*R* ^2^			Classification		
			Run1	Run2	Run3	Run1	Run2	Run3
Cycloheximide	Right	>80	0.95	0.99	0.99	N(FP)	N(FP)	N(FP)
Actinomycin D	Right	>80	0.93	0.98	0.98	N	N(FP)	N(FP)
Ketoconazole	Right	>80	0.97	0.93	0.96	N(FP)	N(FP)	N(FP)
Fulvestrant	Right	>80	0.97	0.9	0.98	N(FP)	P*a*	N(FP)*b*
Norethindrone acetate	Left	>80	0.85	0.73	0.62	Not P due to shift in the opposite direction		

Abbreviations: $S_{c}^{n}$, normalized dose response for the specificity control; N, negative; FP, false positive; P, positive.

Application of the data interpretation criteria results in a FP to be categorized as N.

a Due to borderline value of *R*^2^, the outcome could be P or N(FP).

b With removal of the outlier, *R*^2^ changed from 0.7 to 0.98 leading to N(FP).

Of particular interest was the response of Norethinodrone acetate with a shift of the specificity dose response curve to the opposite direction (left shift instead of right shift versus the standard dose response, see Figure 1B). The *R*^2^ < 0.9 would indicate a competitive antagonist but the shift reveals an unusual result. In this situation of a shift to the opposite direction, the *R*^2^ parameter should not be used to make conclusions. Chemicals displaying this type of left-shifted response have been described as well for the Tox21 lux assay. Sixty-five chemicals were found to have a potency shift in the opposite direction (Kleinstreuer *et al.*, 2017).

Given the particular cases of Norethinodrone acetate, it was concluded that the evaluation of the *R*^2^ value shall best be supplemented with a visual inspection and expert judgment of the dose response shift. Given the proven importance of the specificity test, the normalized specificity control response ($S_{c}^{n}$) and the calculation of *R*^2^ were included in the classifier of the AR-CALUX^®^ method (Supplementary Table 2).

#### Relevance and Accuracy Assessment

Relevance of the results obtained was assessed for 20 chemicals tested by all 4 labs and an additional 24 chemicals for which data were obtained by 1 lab only. The AR-CALUX^®^ VMT defined criteria for the classification (Milcamps *et al.*, 2020) leading to the results depicted in Table 7. The relevance was evaluated on the basis of comparing the mean of the classifications obtained per chemical with classifications reported with other ARTA methods, the AR-reference list, relevant Toxcast assays, and the AR-pathway model (Table 7). Often the compiled classifications were concordant but differences were also noted.

**Table 7.** Comparison of Obtained Classifications of the AR-CALUX^®^ Method with Other ARTA Methods, the AR-Reference List, Relevant Toxcast Assays, and the AR-Pathway Model

	AR-CALUX ^®^	AR REF	AREco-screen™	22Rv1/MMTV/GR-KO TA	Tox21 Luc	ToR21 Bla	AR path-way	AR-CALUX^®^	AR REF	AR-Eco-screen™	22Rv1/MMTV/GR-KO TA	Tox21 Luc	Tox21 Bla	AR Path-way
Test Chemical	Agonist							Antagonist						
17β-Trenbolone	P	**P**			P	P	P	N				N	P	N
Stanozolol	P	**P**						N				N		
Spironolactone	N				P	P	N	P	**P**			P	P	ID
Medroxyprogesterone acetate	P	**P**	P	P	P	P		N			N	N	P	
Bisphenol A	N		N	N	N	N	N	P	**P**	P	P	P	P	P
Bicalutamide	N			N	N	N	N	P	**P**		P	P	P	P
Tamoxifen	N	**N**			N	P	N	N				P	ID	N
Atrazine	N	**N**		N	N	N	N	N	**N**	N	N	N	N	N
Vinclozolin	N			N	N	N	N	P	**P**	P	P	P	P	P
Prochloraz	N	**N**		N	N	N	N	P	**P**	P	P	P	FP	P
Fluoxymesterone	P	**P**			P	P		N				N	N	
Benzylbutyl phthalate	N	**N**	N	N	N	N	N	P			P	P	N	N
Hydroxyflutamide	N		N	P	P		N	P	**P**	P	P	P	P	P
Levonorgestrel	P	**P**		P	P	P	P	N			N	N	P	N
Cyproterone acetate	N	P*			P	N	P	P	**P**			P	P	P
Nandrolone (19-Nortestosterone)	P	**P**			P	P		N				N	N	
o, p’-DDT	N	**N**		N	N	N	N	P	P *		P	P	FP	P
Methyltrienolone (R1881)	P	**P**						N						
Norethindrone acetate	P	**P**			P	P		ID				ID	P	
Linuron	N			N	N	N	N	P	**P**		P	P	P	P
Methyltestosterone	P	**P**		P	P	P	P	N	**N**		N	N	N	N
Norethindrone	P	**P**			P	P	P	N				N	P	N
Mifepristone	N				P	N	N	P	**P**			P	P	ID
Disulfiram	N				N	P	N	N				FP	FP	FP
17α-Ethynyl estradiol	N		N	P	N	P	N	P			P	P	P	P
Sodium azide	N				N	N	N	N				N	N	N
Diethylhexyl phthalate	N		N	N	N	N	N	N		N	N	N	N	N
Methyldihydrotestosterone	P		P	P	p	P		N		N	N	ID	N	
17β-Estradiol	P		P	P	P	P	P	P			P	P	FP	N
Propylthiouracil	N			N	N	N	N	N		N	N	N	N	N
Phenolphthalin	N				N	N	N	N				N	N	N
2,4,5-Trichlorophenoxyacetic acid	N				N	N	N	N				N	N	N
Actinomycin D	N				N	N		N(FP)				FP	P	
Diethylstilbestrol	N				N	N	N	P				P	FP	P
L-Thyroxine	N				N	N		N				N	N	
Haloperidol	N				N	N	N	N				FP	N	ID
Pimozide	N				N	N		N				ID	FP	
Progesterone	N			P	P	P	P	P			P	FP	N	FP
2-sec-Butylphenol	N				N	N	N	P				N	N	N
Corticosterone	N			P	P	P	P	P			P	N	N	N
Ketoconazole	N				N	P	N	N(FP)				FP	FP	FP
Finasteride	N				N	N	N	P				P	P	P
Fulvestrant	N				N	N	N	N(FP)				P	P	N
Cycloheximide	N				N	N	N	N(FP)				FP	N	FP

Grey shading indicates consistent classification of the AR-CALUX^®^ with the AR-Reference list and/or the other 2 ARTAs; in bold shows the AR-reference list; boxed classification in the 22Rv1 TA, not consistent with AR-CALUX^®^; AR REF, AR-reference list of ICCVAM; FP, false positive; P*, weak response; ID, indeterminate; blank space, not tested.

*Comparison to the ICCVAM AR-Reference list:* Of the 44 chemicals tested with the AR-CALUX**^®^** method, 24 could be compared to the AR-Reference list and 23 gave a concordant classification for agonist or antagonist properties (Table 7). An identical classification was observed for all with exception of Cyproterone acetate in the agonist assay. This chemical was scored negative with the AR-CALUX^®^ method and reported as weak positive in the AR-Reference list. The different scoring in the AR-CALUX^®^ validation study is likely due to the concentration applied, being 3 µM as the highest nontoxic concentration. Sonneveld *et al.*, (2005) reported AR agonism for this chemical with the AR-CALUX^®^ cell line at a relatively high concentration (EC_50_ of 4 µM).

*Comparison to results of the mechanistically similar methods AR-EcoScreen™ and the 22Rv1/MMTV GR-KO TA*: Of 44 chemicals tested with the AR-CALUX**^®^** method, 12 and 22 respectively could be compared with the 2 validated ARTA methods (AR-EcoScreen™ and 22Rv1/MMTV/GR-KO TA) (Table 7). A 100% concordance was found with the results from the validated AR-EcoScreen™ method for both agonist and antagonist assay. In comparison to the recently validated 22Rv1/MMTV/GR-KO TA, 100% concordance was found for results obtained in the antagonist assay but a few differences were noted for the agonist assay results (55.5% positive concordance). This included Hydroxyflutamide and 17 Ethynyl estradiol for which the results were concordant between the AR-CALUX^®^ and AR-EcoScreen™ methods (negative) but not with the 22Rv1/MMTV/GR-KO TA (positive). Progesterone and Corticosterone have different classifications between the AR-CALUX^®^ (negative) and the 22Rv1/MMTV/GR-KO TA (positive). These chemicals were not tested within the AR-EcoScreen™ validation study but Corticosterone was reported in the publication of Araki *et al.* (2005) as negative. The few differences observed with the 22Rv1/MMTV/GR-KO TA could be attributed to the difference in cell line but possibly also to the concentrations of chemical tested.

The chemical 17β-Estradiol, known as an ER agonist, shows AR agonist activity with all 3 test methods although in the AR-CALUX^®^ method only a weak activity was observed. It also shows AR antagonist response with the AR-CALUX^®^ method and the 22Rv1/MMTV/GR-KO TA. Activity as both antagonist and agonist is an indication of selective androgen receptor modulation, also known as SARM. SARMs are chemicals that activate their cognate receptor in particular target tissues without affecting other tissues. They have been reported also for other methods such as the YAS assays (Bovee *et al.*, 2010).

*Comparison to the compiled classifications for the 2 Tox21 ARTA assays (Tox21Luc and Tox21Bla), and the AR pathway computational model*: The data for these sources are publicly available and the classifications depend on individual evaluation and expert judgment (Kleinstreuer *et al.*, 2017; US EPA, 2019). The different data sources did not always yield concordant results as could be expected. The classifications reported in this article depend on expert evaluation for the ToxCast assays by the VMG AR-CALUX^®^ and can therefore have a subjective bias. For a few chemicals, the available data were limited. Caution is therefore advised with the interpretation of the data in these later 3 lists. Nevertheless, these data provide some useful reference points for overall comparison and information.

Performance values were calculated based on the ICCVAM AR-Reference list (2017) given that this list is the most reliable (peer-reviewed, published) source of *in vitro* data (Table 8). It covers a range of potencies, both strong and weak (ant)agonists. The AR-Reference list does not always provide information on both agonist and antagonist behavior, leading to 16 test chemicals that could be compared for agonist behavior, and, 12 for antagonist behavior. Positive and negative concordance for both agonist and antagonist classifications were 100% except for 1 chemical that had been scored differently in the AR-CALUX^®^ antagonist assay (Cyproterone acetate, evaluated by 1 lab only, see also above). Performance values calculated based on comparison to the 2 other ARTAs show 100% concordance with the AR-EcoScreen™ for both agonist and antagonist results, whereas concordance was 55% and 100% with the 22Rv1/MMTV/GR-KO TA for agonist respectively antagonist results.

**Table 8.** Performance Values

Agonist									Antagonist							
AR-Reference	Lab 1		Lab 2		Lab 3		Lab 4		Lab 1		Lab 2		Lab 3		Lab 4	
	P	N	P	N	P	N	P	N	P	N	P	N	P	N	P	N
P	10	1	4	0	10	0	6	0	10	0	6	0	6	0	6	0
N	0	5	0	4	0	2	0	1	0	2	0	1	0	1	0	1
Positive concordance	90.9%		100%		100%		100%		100%		100%		100%		100%	
Negative concordance	100%		100%		100%		100%		100%		100%		100%		100%	
Overall concordance	93.8%		100%		100%		100%		100%		100%		100%		100%	

## DISCUSSION

The performance of the method was assessed by EURL ECVAM’s scientific advisory committee (ESAC) (ESAC, 2020) and the reproducibility was concluded to be very good given the almost 100% concordance in classifications when compared to the ICCVAM AR-Reference list. In addition, a low variability was observed for the calculated parameters EC_50_, PC_10_ (agonist assay), IC_50_, and PC_80_ (antagonist assay) (below the 2.5% CV in agonist and antagonist assay). The ICCVAM AR-Reference list, established in 2017, provided a very good basis for comparison given the rigor with which the data were compiled and the decisions made for presenting a reference chemical (Kleinstreuer *et al.*, 2017). Of the 44 chemicals tested with the AR-CALUX**^®^** method, 23 could be compared to the ICCVAM AR-Reference list. Only 1 chemical was classified as negative while in the AR-Reference list it was denoted as a weak positive. The difference in classification is likely to be due to a lower concentration used in the validation study.

The application of a specificity control test, to confirm competitive antagonists, has proven to be useful, both for gaining more confidence that the positive classification is indeed correct, and, for defining false positives. This test considers competitive binding at the AR only and the outcome is defined as either a “true competitive antagonist” or a false positive for the purpose of regulatory decision-making. Though other interferences at the AR level would be of potential interest and would merit further investigation, such has not been the focus of this test. About 20% of the antagonist classifications in the AR-CALUX^®^ validation study were found to be false positives due to the application of the specificity control test. The cause of the non-specific response was likely often due to cytotoxicity (antibiotic or fungicide properties) which was not detected by the accompanying LDH cytotoxicity test or visual inspection in the antagonist assay. For some chemicals, there may be other reasons being false positives such as interference with the AR expression. The validation study showed that a number of actions can be used to arrive at a conclusion for the specificity control test: inspection of the dose response shift, ie, if occurring in the correct direction (right shift), normalization of the specificity dose response, and, the calculation of the criterion *R*^2^. Some caution is advisable with the application of the *R*^2^ as it was found that the shape of the specificity control curve can influence the obtained value. Because the usage of the specificity control test was demonstrated to be quite useful, the result of this test was included in the data interpretation procedure leading to a classification of an antagonist: a non-specific response due to non-competitive binding at the AR was classified as a negative antagonist.

Cytotoxicity testing is an important test in both agonist and antagonist assay of a transactivation assay to exclude responses that are due to cell death. It is especially import for the antagonist assay. For the AR-CALUX^®^ method, it was performed with the LDH kit in analogy with the ER-CALUX^®^ method. It was experienced during the validation study that this test did not add much value as the personal visual inspection of the cells in the wells of the plate proved more sensitive in determining if the cells were showing any sign of stress. The LDH kit may not be the best kit when early signs of death are important to record. Therefore, the updated OECD test guideline TG458 includes the option to use other cytotoxicity kits, which have become available in recent years, as alternatives to the LDH kit.

Of particular interest was the comparison of the performance of the AR-CALUX^®^ method with the 2 other validated ARTA assays, AR-EcoScreen™ and 22Rv1/MMTV GR-KO TA. The AR-CALUX^®^ method performed equally well as the 2 ARTA assays, 1 of which (the AR-EcoScreen™) was already part of an OECD test guideline. For all 3 ARTA methods, the overall variability for the EC_50_ and IC_50_ estimations was below 4%. The classifications for 8 chemicals tested with all 3 methods are concordant for the antagonist assay and also for the 14 chemicals tested additionally in AR-CALUX^®^ and 22Rv1/MMTV GR-KO TA validation studies. With respect to the agonist assay, a few differences were noted between the AR-CALUX**^®^** method and the AR-EcoScreen™ versus the 22Rv1/MMTV/GR-KO TA (55.5% positive concordance and 100% negative concordance). Hydroxyflutamide and 17α-Ethynyl estradiol were found positive with the 22Rv1/MMTV GR-KO TA but not with the other 2 ARTAs; Progesterone and Corticosterone were found positive with the 22Rv1/MMTV GR-KO TA but not with the AR-CALUX^®^ method. Sonneveld *et al.*, (2005), reported that corticosterone had a minor effect in the AR-CALUX^®^ assay (5% RTA in comparison to DHT) when tested at high concentrations (10 µM). A similar observation was made for Progesterone (no EC_50_ reached and RTA of 36%). The differences observed between the 22Rv1/MMTV/GR-KO TA and the 2 other ARTAs could be attributed to different concentrations tested in the 22Rv1/MMTV/GR-KO TA. Differences in cell lines may also be contributing factors such as a different metabolism. Some chemicals may require metabolism for activity or are rendered negative by metabolism. In the AR-CALUX^®^ cell line, a low metabolic activity was shown via RNA sequencing where major classes of metabolic genes were targeted and found to have no or low expression (personal communication from BDS). By incorporating S9 fraction into the method, the impact of metabolism on test chemical activity can be studied (van Vugt-Lussenburg *et al.*, 2018). This is the subject of an ongoing study.

Successfully validated *in vitro* methods with a clear regulatory purpose may become test guidelines (TG) at OECD. The AR-CALUX^®^ method was recently added to the existing TG458, joining the AR-EcoScreen™ already present in the TG458 and simultaneously including the 22Rv1/MMTV GR-KO TA which was also recently validated (Park *et al.*, 2021). This TG, now comprising 3 mechanistically similar ARTA methods, was recently adopted and published (OECD, 2020b). The body of the TG provides an overview of the main characteristics of the 3 methods (eg, type of cell line, reference, and control chemicals, need for a licence agreement, etc.) as well as an overview of the acceptance criteria and terminologies. As such, the end-user has a summary and can make an informed decision on the choice of method to implement. The TG also lists the set of proficiency chemicals and the range of values of important parameters to be calculated. This set of parameters is to be used by any user at the onset of implementing the method of choice, showing proficiency by obtaining the indicated values.

In conclusion, the AR-CALUX^®^*in vitro* method performs well and is a robust method for the categorization of (ant)agonism of the androgen receptor. The test method can be used for screening purposes, mechanistic studies, and hazard assessment, as well as for generating supporting information for regulatory prioritization and decision-making. It could be useful as well as part of a battery of tests to perform non-animal risk assessments. The current methods in TG458 are based on simply identifying chemicals in a dichotomous manner (positive or negative) irrespective of the potency information. This neglects the information that can be deduced from the dose response data as acknowledged by ESAC in their peer-review of the method (ESAC, 2020). The potency information could be useful input for more quantitative approaches towards hazard and risk assessment, in the context of Integrated Approaches to Testing and Assessment (IATA), Defined Approaches (DAs), quantitative Adverse Outcome Pathways (AOPs), Quantitative Structure-Activity Relationships (QSARs), and *in silico* modeling.

## SUPPLEMENTARY DATA

Supplementary data are available at *Toxicological Sciences* online.

## Supplementary Material

kfab078_Supplementary_Data
