# Supplementary material for: Reliability of the AR-CALUX®In Vitro Method Used to Detect Chemicals with (Anti)Androgen Activity: Results of an International Ring Trial
Source: Toxicol Sci. 2021 Jun 24;184(1):170–82. doi: 10.1093/toxsci/kfab078 (PMC8557474; doi:10.1093/toxsci/kfab078)
Supplement: kfab078_Supplementary_Data [file kfab078_supplementary_data.docx]

**Supplementary Material**

**Reliability of the AR-CALUX® *in vitro* method used to detect chemicals with (anti)androgen activity: results of an international ring trial**

Anne Milcamps, Roman Liska, Ingrid Langezaal, Warren Casey, Matthew Dent, Jenny Odum

**Table S1**: Overview of the chemicals used in the AR-CALUX® validation study (reference, control and test chemicals; utility in the various parts of the study).

| **Chemical name** | **CAS** | **Part 1 (Transfer)** | **Part 2**  **(WLR, BLR, Relevance)** | **Part 3**  **(Relevance)** |
| --- | --- | --- | --- | --- |
| 5α-Dihydrotestosterone | 521-18-6 | REF Agonist | REF Agonist | REF Agonist |
| Flutamide | 13311-84-7 | REF Antagonist | REF Antagonist | REF Antagonist |
| Corticosterone | 50-22-6 | NC Agonist | NC | NC / x |
| Levonorgestrel | 797-63-7 | NC Antagonist | NC | NC / x |
| Methyl testosterone | 58-18-4 | PC Agonist | PC | PC / x |
| Linuron | 330-55-2 | PC Antagonist | PC | PC / x |
| Testosterone | 58-22-0 | x |  |  |
| 4-Androstenedione | 63-05-8 | x |  |  |
| Procymidone | 32809-16-8 | x |  |  |
| p,p’-Methoxychlor | 72-43-5 | x |  |  |
| Di-n-butyl phthalate | 84-74-2 | x |  |  |
| Sodium azide | 26628-22-8 | x | x |  |
| 17β-Trenbolone | 10161-33-8 |  | x |  |
| Medroxyprogesterone acetate | 71-58-9 |  | x |  |
| Stanozolol | 10418-03-8 |  | x |  |
| Spironolactone | 52-01-7 |  | x | X |
| Bisphenol A | 80-05-7 |  | x |  |
| 17α-Ethinyl estradiol | 57-63-6 |  | x |  |
| Bicalutamide | 90357-06-5 |  | x |  |
| Tamoxifen | 10540-29-1 |  | x |  |
| Atrazine | 1912-24-9 |  | x |  |
| Disulfiram | 97-77-8 |  | x |  |
| Fluoxymestrone | 76-43-7 |  | x |  |
| Methyldihydrotestosterone | 521-11-9 |  | x |  |
| 17β-Estradiol | 50-28-2 |  | x |  |
| Hydroxyflutamide | 52806-53-8 |  | x |  |
| Vinclozolin | 50471-44-8 |  | x |  |
| Prochloraz | 67747-09-5 |  | x |  |
| Propylthiouracil | 51-52-5 |  | x |  |
| Diethylhexyl phthalate | 117-81-7 |  | x |  |
| Butylbenzyl phthalate | 85-68-7 |  | x |  |
| Progesterone | 57-83-0 |  |  | x |
| Mifepristone | 84371-65-3 |  |  | x |
| Cyproterone acetate | 427-51-0 |  |  | x |
| Methyltrienolone (R1881) | 965-93-5 |  |  | x |
| Norethinodrone | 68-22-4 |  |  | x |
| Norethinodrone acetate | 51-98-9 |  |  | x |
| 19-Nortestosterone | 434-22-0 |  |  | x |
| ICI 182,780 (Fulvestrant) | 129453-61-8 |  |  | x |
| Pimozide | 2062-78-4 |  |  | x |
| Actinomycin D | 50-76-0 |  |  | x |
| Diethylstilbestrol | 56-53-1 |  |  | x |
| Ketoconazole | 65277-42-1 |  |  | x |
| Cycloheximide | 66-81-9 |  |  | x |
| o,p’-DDT | 789-02-6 |  |  | x |
| Finasteride | 98319-26-7 |  |  | x |
| L-Thyroxine | 51-48-9 |  |  | x |
| Haloperidol | 52-86-8 |  |  | x |
| Phenolphthalin | 81-90-3 |  |  | x |
| 2-sec-Butylphenol | 89-72-5 |  |  | x |
| 2,4,5-Trichlorophenoxyacetic acid | 93-76-5 |  |  | x |

X = used as test chemical, NC=negative control, PC=positive control

**Table S2**: Data interpretation criteria (classifier)

**Agonism:** For each run, a test item is considered

A. **Positive** when the relative induction (Y_c_) of the test item is ≥ 10% (REF RPC_10_) for two or more consecutive concentrations.

B. **Negative** in all other cases

**Antagonism:** For each run, a test item is considered

A. **Positive** (competitive antagonist) when the relative induction (Y_c_) of the test item is ≤ 80% (REF RPC_80_) for two or more consecutive concentrations and

Either

- the relative induction of the test items normalised specificity control $\text{s}_{c}^{n}$ > 80% at all concentrations

or when the following two conditions are met:

- the relative induction of the test items normalised specificity control at the highest concentration $\text{s}_{c_{8}}^{n}$is ≤ 80%,
- the square of the correlation coefficient (R^2^) is ≤ 0.9 between the relative induction of the test item (Y_c_) and its specificity control (S_c_)

B. **Negative** in all other cases

**Table S3**: Solubility data obtained for 20 tested chemicals

| **Solubility results of all 4 laboratories** | | | | | | | | | | |
| --- | --- | --- | --- | --- | --- | --- | --- | --- | --- | --- |
| **Solubility in DMSO [mg/ml]** | | | | |  | **Solubility in medium [ug/ml]** | | | | |
| **Chem ID** | Lab 4 | Lab 2 | Lab 1 | Lab 3 |  | Lab 4 | Lab 2 | Lab 1 | Lab 3 |  |
| 17β-Trenbolone | 50 | 50 | 50 | 50 |  | 5 | 50 | 15 | 50 |  |
| Stanozolol | 50 | 50 | 5 | 15 |  | 5 | 5 | 5 | 2 |  |
| Spironolactone | 50 | 50 | 15(15) | 50 |  | 50 | 50 | 15(15) | 50 |  |
| Medroxyprogesterone acetate | 5 | 15 | 1.5 | 15 |  | 5 | 5 | 1.5 | 15 |  |
| Bisphenol A | 50 | 50 | 50 | 50 |  | 50 | 50 | 15 | 50 |  |
| Bicalutamide | 50 | 50 | 50 | 50 |  | 50 | 50 | 50 | 50 |  |
| Disulfiram | 50 | 50 | 50 | 50 |  | 15 | 15 | 5 | 15 |  |
| Tamoxifen | 15 | 15 | 5 | 15 |  | 5 | 15 | 5 | 15 |  |
| Atrazine | 5 | 50 | 15 | 50 |  | 5 | 15 | 15 | 15 |  |
| 17α-Ethynyl estradiol | 50 | 50 | 15 | 50 |  | 15 | 15 | 15 | 15 |  |
| Sodium azide | 15 | 15 | 15 | 15 |  | 5 | 15 | 15 | 15 |  |
| Diethylhexyl phthalate | 50 | 50 | 15 | 50 |  | 50 | 15 | 15 | 50 |  |
| Methyldihydrotestosterone | 15 | 5 | 5 | 15 |  | 5 | 5 | 5 | 5 |  |
| Vinclozolin | 50 | 50 | 50 | 50 |  | 15 | 15 | 5 | 15 |  |
| Prochloraz | 50 | 50 | 50 | 50 |  | 50 | 50 | 50 | 50 |  |
| Fluoxymesterone | 50 | 5 | 50 | 50 |  | 15 | 5 | 5 | 5 |  |
| 17β-Estradiol | 50 | 50 | 50 | 50 |  | 5 | 15 | 5 | 15 |  |
| Benzylbutyl phthalate | 50 | 50 | 50 | 50 |  | 15 | 50 | 1.5 | 15 |  |
| Propylthiouracil | 50 | 50 | 50 | 50 |  | 50 | 50 | 50 | 50 |  |
| Hydroxyflutamide | 50 | 50 | 1.5 | 50 |  | 50 | 50 | 1.5 | 50 |  |

Results shown are from solubility detected by both LDH test and visual checking. Grey shading indicates the

same maximal solubility detected in all four labs

|  |  |
| --- | --- |
|  |  |

Panel A: Relative Induction for DHT obtained per lab (all accepted runs).

|  |  |
| --- | --- |
|  |  |

Panel B: Relative Induction for Flutamide (standard response and specificity control) per lab (all accepted run). Black full line presents the standard antagonist response, blue dashed line the response for the specificity control. Horizontal line indicates the thresholds for classification

**Figure S1:** Visual presentation of the dose responses for reference chemicals DHT (agonist assay) and Flutamide (antagonist assay and specificity control).

|  |  |  |
| --- | --- | --- |
|  |  |  |
|  |  |  |
|  |  |  |

|  | | |  | |  | | |
| --- | --- | --- | --- | --- | --- | --- | --- |
|  | | |  | |  | | |
|  | | |  | |  | | |
|  | | |  | |  | | |
|  | | |  | |  | | |
|  | |  | | |  | |  |
|  | |  | | |  | |  |
|  | |  | | |  | |  |
|  | |  | | |  | |  |
|  | |  | | |  | |  |
|  | |  | | |  | |  |
|  |  | | |  | |  |  |
|  |  | | |  | |  |  |

**Figure S2**: Visual presentation of the dose responses (relative induction) for 44 chemicals tested with the AR-CALUX® agonist assay as obtained in the validation study. Lab 3 (
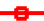
), Lab 2 (
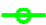
), Lab 1 (
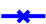
) and Lab 3 (
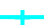
 ). Black dashed horizontal line indicates the 10% threshold for classification. Chemicals with a positive response are shown up-front.

|  |  |  |
| --- | --- | --- |
|  |  |  |
|  |  |  |
|  |  |  |
|  |  |  |

|  | |  | |  | |
| --- | --- | --- | --- | --- | --- |
|  | |  | |  | |
|  |  | |  | |  |
|  |  | |  | |  |
|  |  | |  | |  |
|  |  | |  | |  |
|  |  | |  | |  |
|  |  | |  | |  |
|  |  | |  | |  |
|  |  | |  | |  |
|  |  | |  | |  |
|  |  | |  | |  |

**Figure S3**: Visual presentation of the dose responses (relative induction) for 44 chemicals tested with the AR-CALUX® antagonist assay as obtained in the validation study. In this presentation, the dose responses from the specificity control have not been normalised in order to show the distinction between the responses with or without excess of the ligand DHT. Lab 3 (
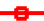
), Lab 2 (
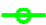
), Lab 1 (
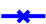
) and Lab 3 (
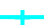
 ). Full line presents the standard antagonist response, dashed line corresponds to response of the specificity control, if applicable. Black dashed horizontal line indicates the 80% threshold for classification. Chemicals with a positive response are shown up-front, followed by those with a negative response. The latter five were concluded as false positives.


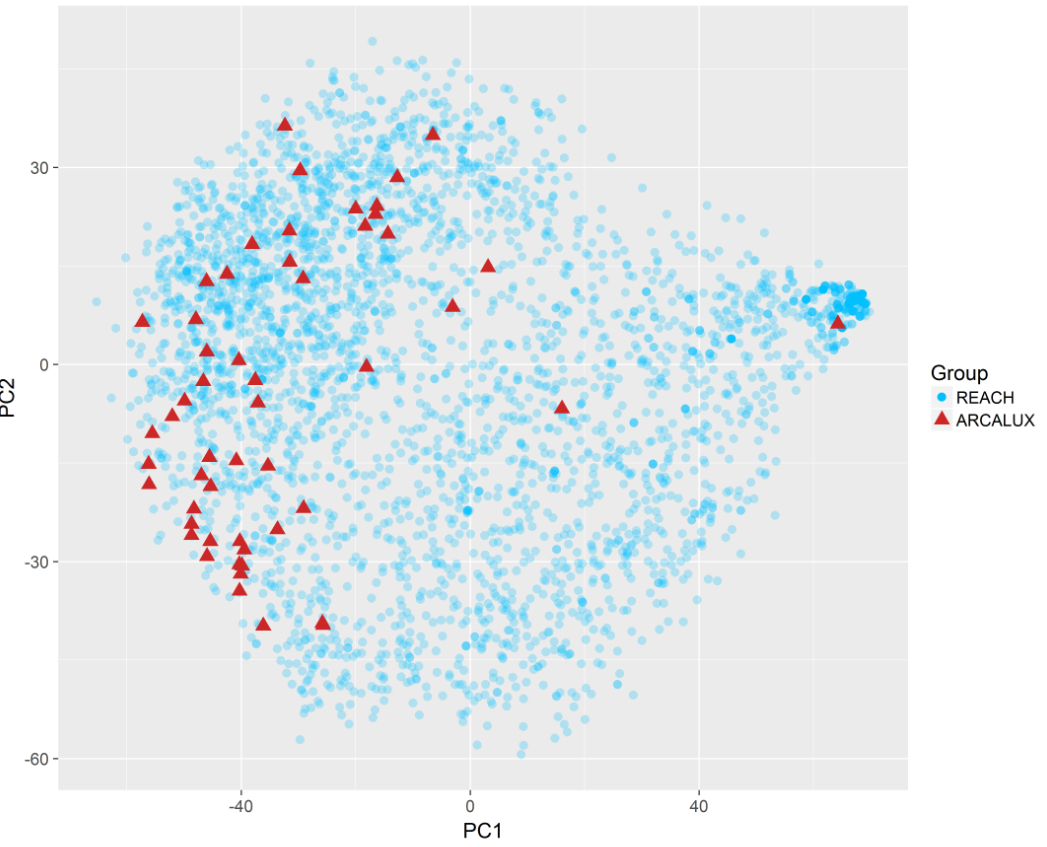


**Figure S4A**: Principal Component Analysis calculated on the structural profiles (Tanimoto index) of the REACH chemicals versus AR-CALUX® validation set. The axis and positions of the chemicals correspond to the first two principal components of the similarity matrix of the chemicals built using the RDKit (Landrum G. RDKit: Open-source informatics. 2015. http://www.rdkit.org) atomic pairs fingerprints. In blue: REACH chemicals; in red: AR-CALUX® validation set.


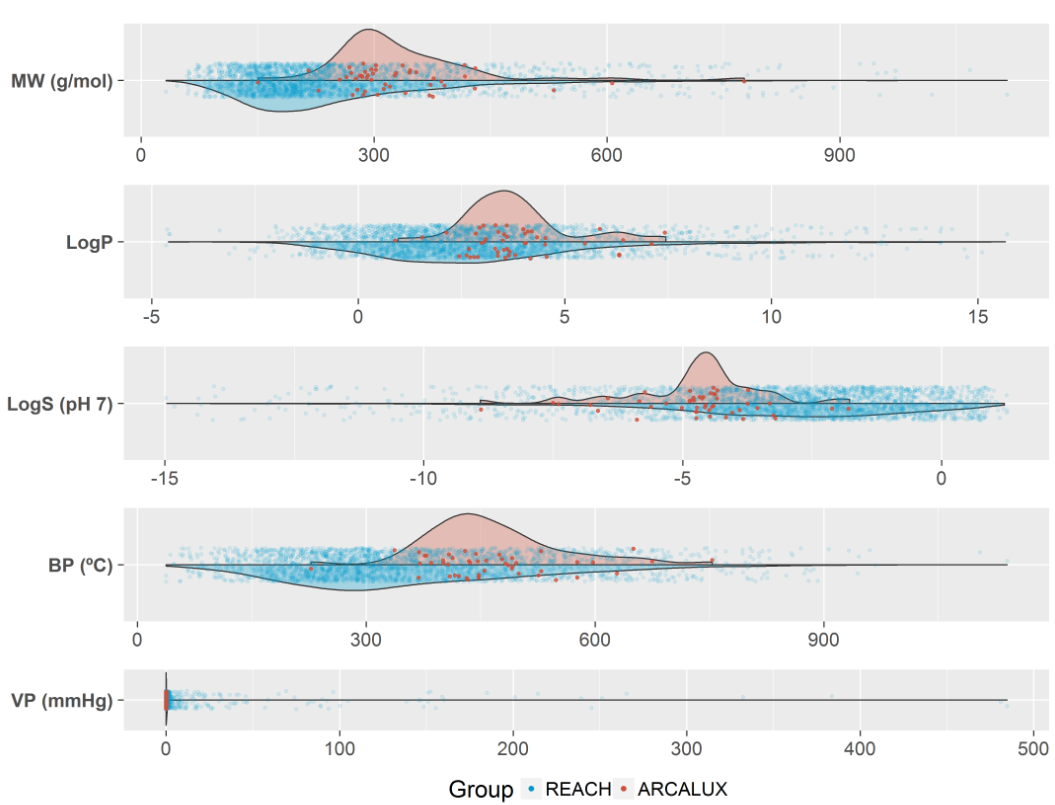


**Figure S4B**: Distribution of physicochemical properties of the AR-CALUX® validation set versus the REACH chemicals. In blue: REACH chemicals, in red: AR-CALUX® validation set. The physicochemical predictions were carried out with ACDLabs/Perceptra.
